# Mechanisms of reproductive isolation between incipient species in the *Anopheles gambiae* species complex

**DOI:** 10.1186/1475-2875-13-S1-O4

**Published:** 2014-09-22

**Authors:** Roch Dabiré, Simon Sawadogo, Abdoulaye Diabaté, Hyacinthe Toé, Pierre Kengne, Ali Ouari, Carlo Costantini, Clement Gouagna, Cedric Pennetier, Frederic Simard, Thierry Baldet, Tovi Lehman, Gabriella Gibson

**Affiliations:** 1 Institut de Recherche en Sciences de la Santé (IRSS)/Centre Muraz, Bobo-Dioulasso, Burkina Faso; 2 Institut de Recherche pour le Developpement (IRD), Montpellier, France; 3 Centre de Cooperation Internationale en Recherche Agronomique pour le Developpement (CIRAD), Cotonou, Benin; 4 Laboratory of Malaria and Vector Research (NIAID/NIH), Rockville, MD, USA; 5 Natural Resources Institute, University of Greenwich, Chatham, Kent, UK

## 

One of the greatest challenges in reducing malaria transmission in sub-Saharan Africa is the high degree of plasticity in the behaviour and ecology of members of the *Anopheles gambiae* complex, which includes the most efficient malaria vectors globally. For example, in areas of West Africa, sympatric populations of the M and S molecular forms of *An. gambiae s.s.* have diverged into recognized species (*An. coluzzii* Coetzee & Wilkerson sp.n. and the nominative species, *An. gambiae s.s*., respectively) through ecological speciation. Whereas the main breeding sites for *An. gambiae s.s*. continue to be temporary water bodies, *An. coluzzii* has become adapted to breeding well in irrigated fields, which has led to increases in relative abundance and duration of breeding seasons, and, hence, an enhancement of malaria transmission throughout the year. Thus, an increase in malaria prevalence is associated with a process of speciation that gave rise relatively rapidly to a new vector species highly adapted to a new breeding habitat created by changes in agricultural practice.

To face this challenge, it is important that we understand the mechanisms by which this kind of speciation occurs. There is good evidence that speciation in this case has involved reproductive isolation, and, therefore, a five year study of sympatric populations of the M and S molecular forms in the Bobo Dioulasso area of Burkina Faso was undertaken to identify mechanisms that limit cross-matings. Mating in the *An. gambiae* complex generally occurs in swarms consisting mainly of males, which form at specific times of day at consistent sites defined by physical features in the environment, e.g., over wells, piles of wood, clearings in vegetation. More than 1,000 mating swarms were documented; a measurable degree of reproductive isolation could be explained by significant differences between species in seasonal abundance, characteristics of swarm sites and daily timing (circadian rhythm) of swarming. Although these factors each contribute to a reduced likelihood that males and females of ‘opposite’ molecular form encounter each other at close range in a mating swarm, both species were occasionally found in mixed swarms. All of the females caught in mixed swarms, however, had been inseminated by a conspecific male. Hence, further research is required to establish the close-range mechanism(s) of species recognition that seem to occur within swarms, such as auditory communication, that lead to assortative mating.

